# Key actors in driving behavioural change in relation to on-farm biosecurity; a Northern Ireland perspective

**DOI:** 10.1186/s13620-018-0125-1

**Published:** 2018-06-14

**Authors:** A. Lahuerta-Marin, M. L. Brennan, G. Finney, M. J. H. O’Hagan, C. Jack

**Affiliations:** 1 Bacteriology Branch, Veterinary Sciences Division, Agri-Food and Biosciences Institute (AFBI), Stoney Road, Belfast, BT4 3SD UK; 2 Centre for Evidence-based Veterinary Medicine, School of Veterinary Medicine and Science, The University of Nottingham, Sutton Bonington Campus, Leicestershire, LE12 5RD UK; 3 Agri-Food and Biosciences Institute (AFBI) Hillsborough, Large Park, Hillsborough, BT26 6DR UK; 4 Department of Agriculture, Environment and Rural Affairs (DAERA), Veterinary Epidemiology Unit (VEU), Dundonald House, Upper Newtownards Road, Belfast, BT4 3SB UK; 5 Agri-Food and Biosciences Institute (AFBI), 18a Newforge lane, Belfast, BT9 5PX UK

**Keywords:** Biosecurity, Disease prevention, Animal health, Farmer behaviour, Training, Workshop

## Abstract

**Background:**

Agriculture and farming are valued contributors to local economy in Northern Ireland (NI). There is limited knowledge about farmers’ behaviours and attitudes towards disease biosecurity measures. As part of a larger project, a scenario-based workshop with key stakeholders was organised by the Agri-Food and Biosciences Institute (AFBI)-NI in December 2015.

**Results:**

A total of 22 participants belonging to 12 different institutions took part in the workshop. Participants were presented with an overview of previously conducted biosecurity research in NI and England. In small groups, participants were subsequently asked to discuss and give their opinions about a series of questions across four k*e*y areas in a semi-structured approach with an external facilitator. The key areas were 1- disease risk perception at the farm level; 2-perceived barriers to implementing on farm biosecurity measures; 3- avenues to successful behaviour change and 4-key industry responsibilities and roles. The discussion showed that training in biosecurity for farmers is important and necessary. Training was recommended to be provided by veterinary surgeons, preferably via a face-to-face format. The discussion addressing disease disclosure proved particularly challenging between those who were prospective buyers of cattle, and those who sold cattle.

**Conclusions:**

This workshop provided a unique and invaluable insight into key issues regarding farm level biosecurity activities. From a policy perspective, delivering improved on-farm biosecurity must be addressed via a multidisciplinary approach. This can only be achieved with active involvement, commitment and support of a number of key industry and government stakeholders.

**Electronic supplementary material:**

The online version of this article (10.1186/s13620-018-0125-1) contains supplementary material, which is available to authorized users.

## Introduction

Agriculture makes an important contribution to the Northern Ireland (NI) economy with the sector contributing 1.7% to Gross Value Added in 2017 [1]. The sector is more important in terms of employment relative to agriculture in the UK, with 2.5% of the total civilian workforce employed within the sector compared to 1.1% in the UK. The livestock sectors (dairy, cattle and sheep) make the largest contribution to the gross output of NI agriculture (57%), in contrast to the livestock sector in the UK which contributes 31% to gross output [2]. Livestock farms dominate NI agriculture; the majority of farms are classified as beef and sheep farms (79%) or dairy farms (11%) [2]. In this context, good management of disease prevention at the farm level is critical for the profitability and sustainability of the sector and the agri-food supply chain. A lack of robust controls resulting in disease outbreaks not only has a negative impact at the individual farm level, but also increases the disease threat for surrounding farms and ultimately risks to the wider industry and society [3]. Farm managers and workers in all regions play a pivotal role in shaping and maintaining biosecurity policies to ensure a high animal health status. The effectiveness of such policies will be dependent upon farmers’ behaviours and actions in relation to these biosecurity practices. Research identifies that these behaviours and actions will be influenced by both internal and external factors, individual farmer’s values and their perceptions of risk [4].

Previous studies have shown that farm size, enterprise types, industry structure, geographical location, farm ownership, dependence on farm business income (part-time vs. full-time farmers) can all influence (both positively and negatively) attitudes to farm biosecurity [5–7]. In addition, the farm operator’s age, and level of educational attainment have also been shown to influence biosecurity practices at farm-level. Stakeholder meetings have been used successfully to provide insight and advice to authorities in areas such as the environment and food safety [8]. This approach is particularly beneficial in the case of challenging issues, such as farm biosecurity and behaviour change, which can be difficult to quantify by conventional statistically-driven methods. Therefore, when designing and developing policies around farm level biosecurity implementation, there is a need to understand the motivations and attitudes of the key stakeholders involved, in order to deliver sustained change.

## Approach/Materials and Methods

Incorporated into a wider study exploring attitudes and behaviours around farm level biosecurity, a one-day workshop with a targeted group of key stakeholders (see Additional file 1) from the cattle and sheep sectors in NI was carried out at Grange Farm, AFBI Hillsborough, on 7th December 2015. The main objective of the workshop was to gain an understanding of the behaviours surrounding, and attitudes towards biosecurity implementation by identifying industry led solutions and therefore where government was best placed to provide support.

A range of stakeholders from industry and governmental authorities in NI and Great Britain were invited to attend (*n* = 22). Participants were allocated to a number of small groups (between five and six people), with representatives from each organisation equally distributed amongst the groups. Participants were presented with an overview of previously conducted biosecurity related research in NI and England (see Additional file 2), and were subsequently asked to discuss and give their opinions around a series of questions across four k*e*y areas. The questions were focused around concepts arising from the results and findings of the 2014 AFBI Farm Level Biosecurity Survey (unpublished data). The concepts discussed related to disease/infection risks, barriers to farm-level biosecurity implementation, behaviour change and roles and responsibilities of industry and policy (See Additional file 3). In their groups, participants were given three hours to discuss the relevant issues in a semi-structured approach with an external facilitator (Additional file 4). The results from the group discussions were transcribed and discussed collectively by the authors and summarised as following.

## Results

The workshop facilitated dialogue and communication between the key stakeholders involved in developing, implementing and delivering biosecurity policies and practices at farm level. Four key themes were identified by the authors as a result of the collective discussion.

### Theme 1: How disease/infection risks are perceived at farm level

Results from the AFBI Farm Level Biosecurity Survey revealed that the majority of trading activities among cattle/sheep farmers were carried out locally,- mainly between neighbouring farms or from local markets; there was a generalised impression amongst farmers that the risks from the introduction of disease/infection were perceived to be greater from locations outside of NI. This was reflected in comments such as^1^:“*It’s easier to blame disease as a problem coming from the outside* (i.e. outside of Northern Ireland) *rather than from within”.* The perception that disease/ infection are external not internal has important biosecurity consequences as this diverges from where most risks actually occur, i.e. the most probable way of infection (disease) introduction into the herd/flock will be from local trading activities. Moreover, it was expressed that there was a real need for individual farmers to take ownership of farm level biosecurity and make decisions around implementation of biosecurity measures, *“farmers need to see that it is your farm, it is your animal, it is your animal health*”. Group members expressed that there needed to be a more focused approach to communicating this as it was viewed as an important driver to bringing about improvements in biosecurity at the farm level.

However, there was also acknowledgement that the individual cannot control the collective behaviours of others (i.e. neighbouring farmers), which could result in a frustrated and fatalistic attitude toward disease/infection control.

### Theme 2: The perceived barriers to implementing on farm biosecurity measures

*“Disease is often ‘invisible’ therefore it is difficult to see the result of any actions you take and also you may have a disease present before you know about it”.*

*“Difficult to maintain good neighbour relationships”.*

It was felt that investment in and implementation of biosecurity measures can be challenging due to both Northern Ireland’s traditional system of land rental, namely the conacre system^2^ and also the high prevalence of farms with associated outlying farms [9]. The practicalities of implementing biosecurity practices were described as difficult particularly when they required financial investment. Reflecting on-farm resource pressures, farmers indicated that they would be less likely to implement those biosecurity measures which required large financial investment or place increased demand on farm labour. The newly introduced delivery mechanism for agricultural advisory services in Northern Ireland, ‘Farm Business development (FBD) groups’ were viewed as an important potential mechanism to accomplish and incentivise more on-farm implementation of biosecurity measures. This was given from the perspective that in order to change farmers’ perceptions and behaviours around biosecurity, it was deemed critical that this must be farmer-led but with the support of other key stakeholders and the industry.

### Theme 3: Avenues to successful behaviour change

It was clear from the discussions that there was a perception that education and training could promote behaviour and attitude change: *“Educate farmers about the nature of local versus foreign risks”*

*“Training is about getting the information in the right way to the right farmers”*

*“Take the disease, provide the information on managing the disease and then draw out the biosecurity issues in relation to prevention”*

Local veterinary surgeons were identified as the key drivers in the delivery of biosecurity training at farm-level. Face-to-face training was the preferred delivery system. It was felt that training also needed to be tailored and targeted towards defined groups of farmers with specific needs (e.g. young farmers’ groups or by groups associated with a specific farm enterprise or system type). FBD groups and organised farm visits were identified as key training delivery mechanisms and it was felt that this should be part of a coordinated approach with the support of industry and government. In addition, participants felt that exemplar farmers could play a key role in helping to inform, train and educate other farmers about biosecurity. There was a strong consensus amongst the participants that the focus of training should be on biosecurity relating to non-statutory endemic diseases such as Johne’s disease, including the impacts and costs of disease on their particular farming system and at a wider level. Furthermore it was suggested that farmers would benefit from training in the overarching meaning of ‘biosecurity’ and that new technologies and recording systems might play an important role in helping to assist with farm-level biosecurity planning.

Delegates thought incentives should be used as the key mechanism to deliver behavioural change amongst farmers, although it was envisaged that there would still be a need to monitor and enforce baseline standards through penalties.

### Theme 4: Key industry responsibilities and roles

*“Farmers need to be at the heart of biosecurity training- they should be in the ‘focus of it’-but all other stakeholder groups should be involved- processors, auctioneers and vets.”*

*“Industry needs to embrace biosecurity in a unified way (Ulster Farmers’ Union (UFU), industry (i.e. meat processors/retailers)) and there is a need to identify good practice “real farmers””.*

Participants generally indicated support for a system of informed purchasing in relation to the disease status of animals when being sold, although there was an acknowledgement of some of the issues that may arise: *“Buyers want it, sellers not so keen. Problem is with the legal aspect*”. Importantly, all concurred that this would need a lot of consideration, including exploring issues around data protection and agreement on which diseases should be disclosed. It was felt that the disclosure system must be reliable and trusted and there was a consensus that disease status should be made available on a voluntary basis with a gradual progression to a compulsory system. Participants thought the scheme should have the full support of the industry with all parties along the production and supply chain system having confidence in its capabilities. There was a lack of agreement as to whether such a scheme should apply to the whole farming industry or to certain types of farm.

## Discussion

Although several socio-epidemiological studies have been undertaken to understand on-farm biosecurity at a UK and NI level [10–13], there has been limited exploration around combined farmer and stakeholder attitudes and solutions. This workshop was unique in that farmers and key stakeholders were able to discuss these issues together and express their opinion collectively regarding the importance and relevance of biosecurity implementation to the NI livestock sector. This approach was particularly innovative as it was the first time that such a workshop on biosecurity had been hosted at the Northern Ireland regional level. The results of this workshop have provided a ground breaking first step to establishing the key issues and facilitated an opportunity for key actors to express their views and needs. The main conclusions of the workshop will provide policymakers with an important insight into understanding the attitudes and behaviours around on-farm biosecurity amongst farmers and other key representatives of the industry as well as identification of possible solutions. The discussion showed that training in biosecurity for farmers is important and necessary. Training was recommended to be provided by veterinary surgeons and face-to-face was the preferred format. Moreover, training should be tailor-made to cover the audience needs as highlighted in a recent UK study on veterinary practitioners specialised in dairy cattle [14]. Therefore the veterinary profession needs to be more involved in on farm biosecurity training.

Within the workshop, the discussion around the area of disease disclosure proved particularly challenging and there was clear dichotomy between the views and attitudes of those who were prospective buyers of cattle and those who were selling cattle. In the future, the farming sector will face increased challenges around making the disclosure of disease status of individual farms public, something which has already been implemented recently at cattle markets in Wales in relation to herd status and bovine tuberculosis [15]. Farmers need to take responsibility collectively for undertaking more biosecurity, within the constraints of what is possible bearing in mind some of the traditional structures of farming in NI (e.g. conacre) and the different businesses that individuals have (e.g. buyers and sellers).

The support of the industry and other interested stakeholders will provide assurance and guidance to farmers on the need for implementation of key biosecurity measures despite the cost/labour effort involved. There are some financial issues that must be addressed before widespread biosecurity can be undertaken. There was recognition that both incentives, and penalties, are needed to encourage farmers to get involved, and this was identified as a role for wider industry. In addition, FBD groups may play an important driver in changing local farmer attitudes towards perceived versus real risk of the introduction of disease into the herd/flock.

On the limitations we are aware that there are some recent quantitative studies that have measured the effect on biosecurity practices on key animal diseases [16]. Thus, some of the conclusions from this meeting should be tested in order to measure the real impact of the practical application of biosecurity interventions in herds and flocks in NI in the future.

In summary, the workshop provided a very unique and invaluable insight into some of the current and key issues regarding farm level biosecurity. It has also highlighted that, from a policy perspective, delivering improved on-farm biosecurity must be addressed through a multidisciplinary approach. Although focussed on attitudinal and behavioural change at the farm level, this can only be brought about through the active involvement, commitment and support of a number of key industry and government stakeholders. The challenges of this from a policy perspective are increasingly important given the uncertainties around the UK’s exit from the European Union.

## Additional files

Additional file 1: List of participating institutions. (DOCX 13 kb)

Additional file 2: Agenda of the one-day workshop. (DOCX 244 kb)

Additional file 3: Discussion Themes. (PDF 641 kb)

Additional file 4: Pictures of the one-day workshop on biosecurity AFBI- Hillsborough. (DOCX 1141 kb)

## Abbreviations

AFBI: Agri-Food and Biosciences Institute
DAERA: Department of Agriculture, Environment and Rural Affairs (formerly DARD)
DARD: Department of Agriculture and Rural Development
EFSA: European Food Safety Agency
FBD: Farm Business Development
NI: Northern Ireland
UFU: Ulster Farmers’ Union
UK: United Kingdom

## Footnotes

1 All text under quotation marks and written in italics represent actual statements reordered from participants in the discussions

2 Conacre is a system of renting agricultural land that is unique to Ireland. The term is used to reflect the system where landowners who wish to retain their land but not farm it can licence the land to another farmer, usually for a period of eleven months.
