# Supplementary material for: Key actors in driving behavioural change in relation to on-farm biosecurity; a Northern Ireland perspective
Source: Ir Vet J. 2018 Jun 14;71:14. doi: 10.1186/s13620-018-0125-1 (PMC6001042; doi:10.1186/s13620-018-0125-1)
Supplement: Supplementary file 1 — List of participating institutions. (DOCX 13 kb) [file 13620_2018_125_MOESM1_ESM.docx]

**Additional file 1: List of participating institutions**

| **Institution** |
| --- |
| Agri-Food and Biosciences Institute (AFBI) |
| Northern Ireland Veterinary Association (NIVA) |
| Department of Agriculture, Environment and Rural Affairs (DAERA) |
| School of Veterinary Medicine and Science, Univ. of Nottingham |
| Ulster Farmers Union (UFU) |
| College of Agriculture , Food and Rural Enterprise ( CAFRE)-DAERA |
| Veterinary Epidemiology Unit (VEU)-DARD |
| Cattle Herd Certification Standards (CHeCS) |
| British Cattle Veterinary Association (BCVA) |
| Tuberculosis Strategic Partnership Group (TBSPG) |
| LANTRA |
| The Association of Veterinary Surgeons Practising in Northern Ireland (AVSPNI) |
