# Supplementary material for: Key actors in driving behavioural change in relation to on-farm biosecurity; a Northern Ireland perspective
Source: Ir Vet J. 2018 Jun 14;71:14. doi: 10.1186/s13620-018-0125-1 (PMC6001042; doi:10.1186/s13620-018-0125-1)
Supplement: Supplementary file 2 — Agenda of the one-day workshop. (DOCX 244 kb) [file 13620_2018_125_MOESM2_ESM.docx]

**Additional file 2: Agenda of the one-day workshop**
