# Supplementary material for: Key actors in driving behavioural change in relation to on-farm biosecurity; a Northern Ireland perspective
Source: Ir Vet J. 2018 Jun 14;71:14. doi: 10.1186/s13620-018-0125-1 (PMC6001042; doi:10.1186/s13620-018-0125-1)
Supplement: Supplementary file 3 — Discussion Themes. (PDF 641 kb) [file 13620_2018_125_MOESM3_ESM.pdf]

Agri-Food and Biosciences Institute

Workshop on sheep and cattle farmer's understanding of biosecurity, and their attitudes towards applying biosecurity measures when dealing with disease

AFBI-Hillsborough, 7th December 2015

Discussion Themes

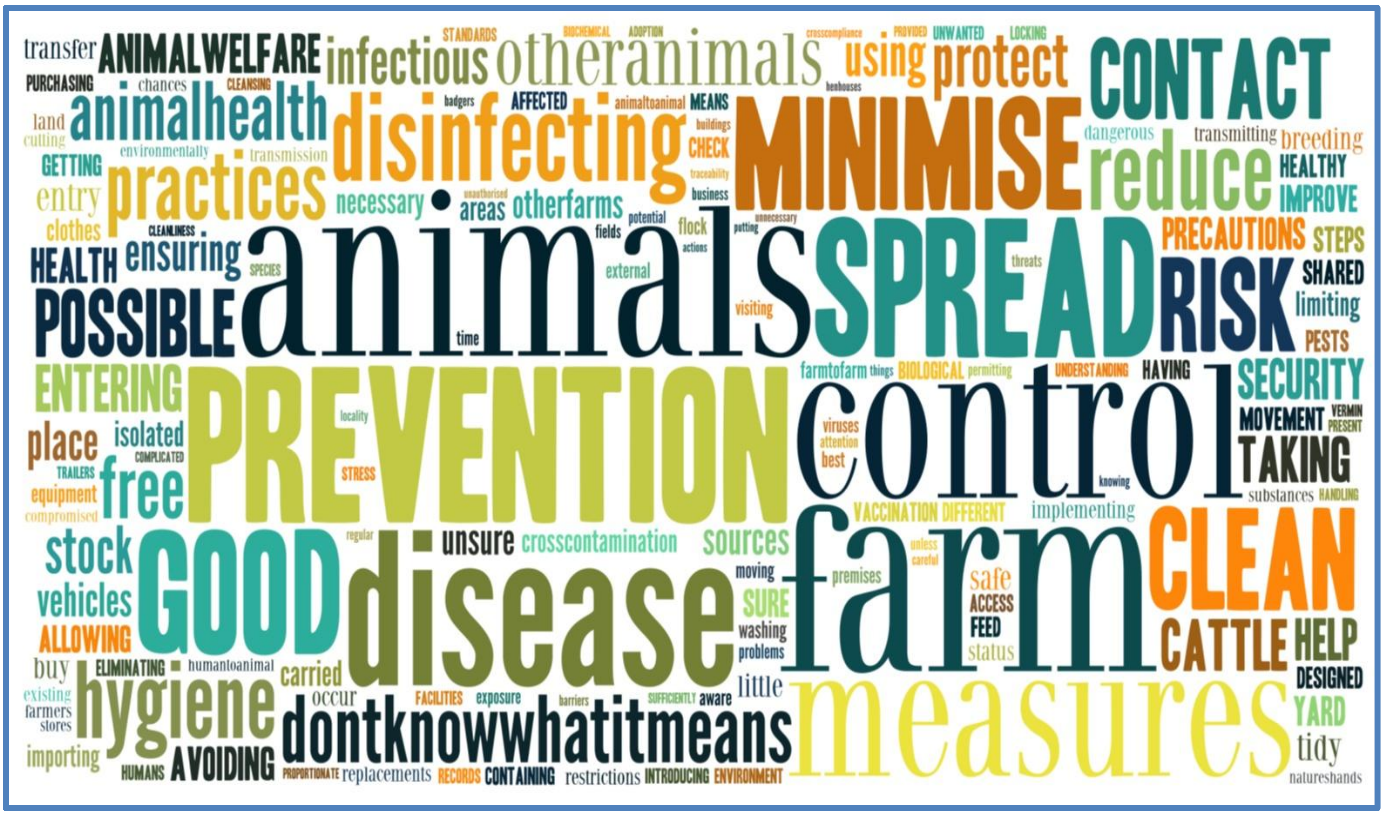

Background

Effective biosecurity at the farm level is extremely important to diminish the risk of the introduction and/or spread of animal diseases. However, in Northern Ireland (NI), there is very limited information about the general practices that cattle and sheep farmers implement and also about their beliefs and attitudes towards biosecurity.

In view that European legislation may request the implementation of biosecurity practices by farm owners in the near future, a DARD sponsored 2014- survey was performed among cattle and sheep farmers in NI to find out more information this subject. The survey was coordinated by AFBI.

The main highlights found in the survey were:

1- The majority of trading among cattle/sheep farmers is done locally- mainly from neighbour farmers or local marts;
2- Cattle and sheep farmers do not seem to perceive major risk on the introduction of disease from local sources but from imported animals outside NI;
3- On animal health checks, the majority of farmers only perform the minimum required by law;
4- Bovine tuberculosis and brucellosis were the top priority diseases for cattle farmers while toxoplamosis and sheep scab were the main concern diseases for sheep farmers;
5- The preferred sources of advice on biosecurity were the private veterinary practitioners, DARD Veterinary officers and DARD website;
6- Farmers were keen on more information and receiving further training on biosecurity. The preferred method of delivery would be "face-to-face";
7- The majority of framers indicated that disclosure of disease status of the farm to be traded with would be a good and useful biosecurity practice;
8- Monitoring and quarantine of sick animals were the initial responses to disease incidents. However, larger farms were less likely to monitor/quarantine their animals in comparison to smaller scale enterprises;
9- Least cost/labour intensive biosecurity practices were the preferred option;
10- Prevention and transmission of disease together with pathogen and animal health were the most common terms used in the definition "Biosecurity"

Purpose and objectives

Stakeholders will be divided upon small groups (5-6 people) to discuss structured questions about some key results from the survey. The answers from each group will be presented in plenary session at the end of the workshop. Each group should designate a chair and a note-taker.

The objective is the generation of ideas, solutions and possible drivers on the subject that would help DARD in the development of policies or advice to farmers.

Theme 1 Farm level Skills and Training- (20 min)

1 The AFBI farm level biosecurity survey indicated that farmers would like more training on aspects of animal health welfare and biosecurity.

a) From an animal health, welfare and biosecurity perspective, what are the key learning and training priorities for farmers?

b) Farmers indicated a preference for group based learning (as opposed to traditional desk based learning) perhaps through demonstration event, farm visits are local group meetings. Who or which organisation do you feel is best placed to deliver this training?

c) What is your opinion on the use of other Alternative ways of delivering the course e.g. online ?

C) Who should decide on the contents and delivery of this training ?- in collaboration with industry?

D) How do you determine that farmers have implemented what they have learnt?

E) Should a basic level of biosecurity training be mandatory for all farmers and how best do you think this could be facilitated.

F) Research has shown that demonstrations of successful implementation of biosecurity measures and their benefits increased the level of uptake. The AFBI survey identified some confusion about what biosecurity actually means and also whether certain biosecurity measures were relevant or appropriate to a farmers particular farming system resulting which can limited uptake of the measures.

G) Is there a role for a more 'targeted approach' to biosecurity advice by delivering it to specific groups of farmer or tailoring the delivery of the information to specific farming system i.e .older farmers, part-time farmers, full-time farmers, hill farmers, beef finishers .

Theme 2 -Animal Health/Welfare and Farm-biosecurity - (30 min)

Farmers indicated that although they can control what they do, they cannot control the actions of their neighbours.

A) Is there a role at an area level for a voluntary farmer led farm-level, health-welfare-biosecurity initiative/assurance scheme which recognises a collaborative approach to maintaining farm-level biosecurity?

B) Is there a need for further research to be undertaken exploring the role/ use of technology in developing software tools for recording and maintaining farm level animal health welfare and biosecurity records?

Farmers indicated that they perceived biosecurity may become more relevant in the near future. However, farmers did indicate that pressures around the level of investment required to implement biosecurity issues (either financial or labour requirement) would impact on their decision to implement.

C) In what ways could government support increased implementation of biosecurity practices and actions e.g. DARD capital grant schemes?

Theme 3- Perception of risk- How farmers perceive the risk in relation to disease- (20 min)

The AFBI survey results indicated that farmers perceived that the main disease risks were from animals brought in from outside NI. However most of the trading (and associated disease risk) is local.

A) What are, in your opinion, the main drivers behind these beliefs/ perception?

B) How do we change this risk perception?

- How to influence behaviour/ belief change?

C) Which stakeholders should take the lead on this?, veterinary surgeons, DARD, UFU?

D) Individual change versus group change?

Theme 4- Disclosure of disease status- (20 min)

Farmers indicated that having knowledge on the disease status of the farm or animals to purchase will be a useful biosecurity practice. In Welsh markets it has been proposed the disclosure of bovine tuberculosis status on animals to be sent to markets. However, this is a complex and sensitive request. The implementation of this issue will request the wide support of DARD, the industry and other interested stakeholders.

A) Are you in favour or not of making disease information status available for livestock buyers?, please give reasons

B) Should it be voluntary or compulsory?

C) How should the issue on data confidentiality be handled? Who should decide upon this issue?

D) Advantages and disadvantages of allowing this scheme

E) Key factors to take into account? Describe an implementation process step by step

Theme 5- What is next? (20 min)

In the context of the issues raised today, and in meeting the objectives of "going for growth", what should the key issues be around farm level biosecurity over the next 5-10 years?
