# Supplementary material for: Key actors in driving behavioural change in relation to on-farm biosecurity; a Northern Ireland perspective
Source: Ir Vet J. 2018 Jun 14;71:14. doi: 10.1186/s13620-018-0125-1 (PMC6001042; doi:10.1186/s13620-018-0125-1)

**Annex 4 Pictures of the one-day workshop on biosecurity AFBI- Hillsborough**

Picture 1- Group picture


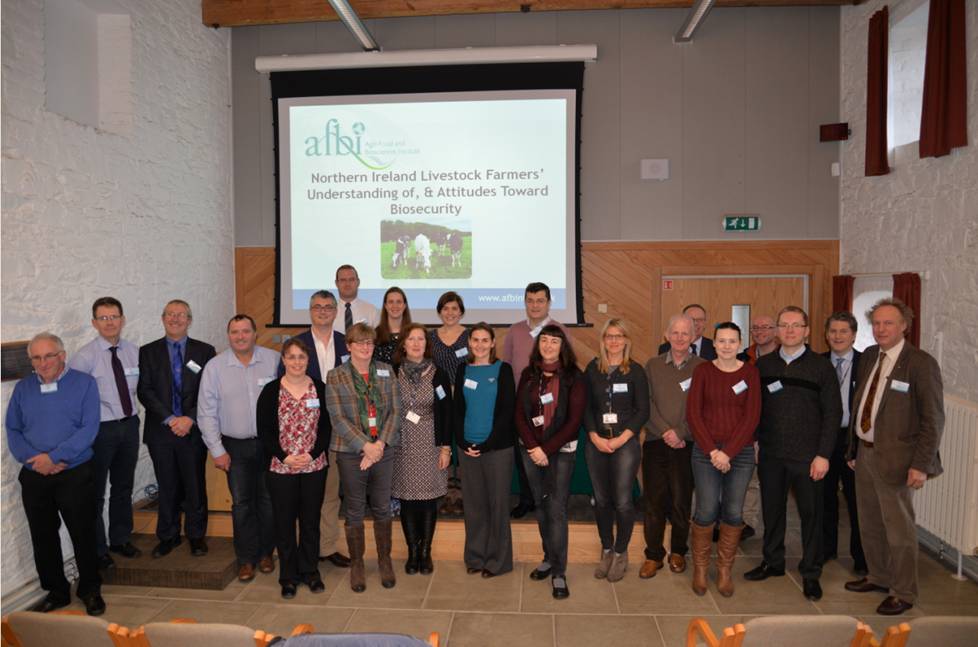


Picture 2- Group discussions


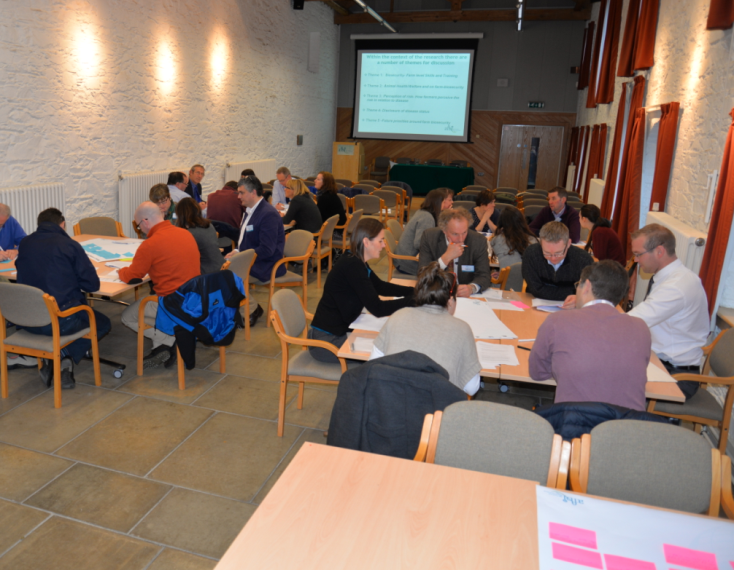


Picture 3- Common discussion and conclusions


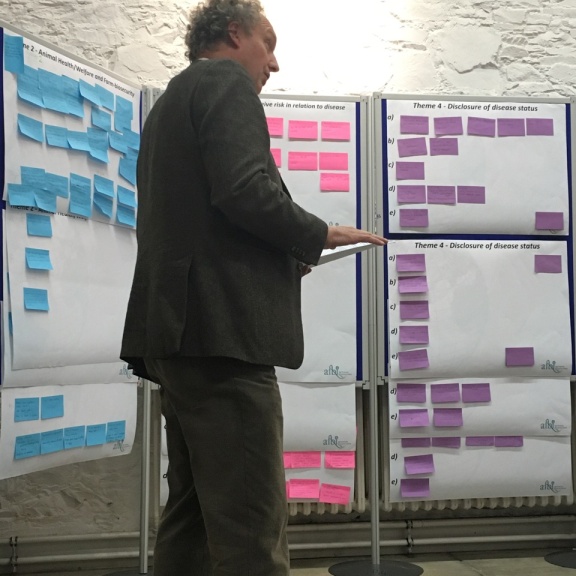

Supplement: Supplementary file 4 — Pictures of the one-day workshop on biosecurity AFBI- Hillsborough. (DOCX 1141 kb) [file 13620_2018_125_MOESM4_ESM.docx]
